# Diagnosis and management of axillary lymph node metastasis from an unknown primary carcinoma: a case report

**DOI:** 10.3389/fonc.2026.1920276

**Published:** 2026-09-10

**Authors:** Motong Xu, Shuo Qiu, Jie Zhou, Yongchun Zhang

**Affiliations:** The Affiliated Hospital of Qingdao University, Qingdao, Shandong, China

**Keywords:** breast cancer, Canhelp^®^-Origin gene expression profiling assay, CUP, GEP, TRPS1

## Abstract

**Background:**

Carcinoma of unknown primary (CUP) represents a heterogeneous group of metastatic malignancies in which the primary tumor site remains unidentified despite comprehensive diagnostic evaluation. Accurate determination of tissue origin is essential for guiding treatment and improving clinical outcomes. The Canhelp^®^-Origin gene expression profiling (GEP) assay, combined with specific immunohistochemical markers, has emerged as a valuable tool for identifying the origin of metastatic carcinomas. However, its clinical application in challenging cases, particularly those with atypical presentation and limited lineage-specific markers, warrants further documentation.

**Case presentation:**

We present a case of a patient with right axillary metastatic carcinoma, in which the primary site remained undetermined after extensive imaging and initial immunohistochemical workup. Integrated application of the Canhelp^®^-Origin GEP assay and immunohistochemical staining for lineage-specific markers, including TRPS1 and SOX10 (+), supported a probable diagnosis of metastatic triple-negative breast cancer, though melanoma and synovial sarcoma could not be fully excluded given overlapping immunophenotype and limited available tissue for additional confirmatory molecular testing. The patient subsequently received an AC-based chemotherapy regimen combined with immunotherapy and sequential radiotherapy. According to RECIST 1.1 criteria, the therapeutic effect was confirmed as complete response (CR), with complete remission of axillary and cervical lymph node metastases. Notably, treatment response is non-specific and cannot serve as retrospective proof of tumor tissue-of-origin.

**Conclusions:**

This case highlights the potential adjunct value of the Canhelp^®^-Origin assay in identifying the tissue of origin in CUP and underscores the supportive diagnostic role of TRPS1 immunohistochemistry in triple-negative breast cancer. These findings demonstrate the clinical utility of an integrated diagnostic strategy for the probabilistic classification of CUP, supporting its potential to guide effective treatment decisions in this challenging clinical setting.

## 1 Introduction

Carcinoma of unknown primary (CUP), a clinical syndrome characterized by a histologically confirmed metastatic malignant tumor, remains undiagnosed in terms of the location of the primary tumor despite a comprehensive clinical assessment. It accounts for 2.3% to 5% of all newly diagnosed cancer cases (1). The absence of an identifiable primary tumor site renders treatment for CUP largely non-targeted, resulting in suboptimal therapeutic outcomes and a generally poor prognosis. Standardized clinical guidelines for CUP management are available from ESMO, NCCN and domestic Chinese expert consensus; nevertheless, high-level clinical evidence for unfavorable-subset CUP remains limited. Accurate identification of the primary lesion enables more precise therapeutic decision-making, thereby improving treatment efficacy and patient survival. The Canhelp^®^-Origin gene expression profiling (GEP) serves as an adjunct molecular classifier in the diagnosis of metastatic carcinoma of unknown primary origin by enabling probabilistic prediction of the tumor’s tissue of origin through comprehensive analysis of its gene expression patterns. In a retrospective study, 71% of molecular test results for tissue-of-origin identification were deemed predictive, based on a similarity score exceeding 45. The breast was the most commonly predicted primary tumor site, and *TRPS1* (Tricho-rhino-phalangeal syndrome type 1) emerged as a reliable biomarker for breast cancer prediction, with an accuracy rate of 75.0% (2).

The immunohistochemical expression of TRPS1 protein exhibits distinct and clinically relevant patterns in breast cancer diagnosis, particularly in the differential diagnosis of triple-negative breast cancer (TNBC) and metastatic carcinomas of unknown primary origin, where it serves as a valuable diagnostic biomarker. However, TRPS1 possesses high sensitivity but limited specificity and is expressed in multiple non-breast tumor entities. In the absence of other specific markers, TRPS1 alone cannot confirm breast origin; multimodal integration is mandatory (3).

This case report presents a case of a right axillary metastatic carcinoma of unknown primary origin. The initial imaging studies and immunohistochemical analysis failed to identify the primary lesion. By organizing origin gene testing in combination with special immunohistochemical indicators such as TRPS1, SOX10, and HER2, multimodal evidence favored the probability of metastatic triple-negative breast cancer, while important differential diagnoses including melanoma and synovial sarcoma could not be formally excluded. After chemotherapy with AC combined with immunotherapy, the axillary and cervical lymph nodes achieved complete remission, and the therapeutic effect was evaluated as complete response (CR) according to the RECIST 1.1 standard. Of note, complete response does not validate tumor tissue origin. This case report further underscores the significant value of TRPS1 protein immunohistochemical expression in the differential diagnosis of metastatic cancer of unknown primary origin, and highlights the critical role of tissue-of-origin gene expression profiling in diagnosing such cancers, offering a novel diagnostic strategy to support precise diagnosis and informed treatment decisions.

## 2 Case report

### 2.1 Patient information and initial presentation

A 61-year-old woman presented to our hospital on December 27, 2024, with a progressively enlarging mass on the right chest wall. The patient sought medical attention due to this symptomatic enlarging lesion, firm in consistency, and poorly demarcated from the surrounding tissues. Her medical history was unremarkable, with no documented family history of malignancies.

### 2.2 Prior surgical intervention and initial pathology

The patient had undergone surgical excision of the lesion at an outside hospital on November 11, 2024. Intraoperative findings revealed a 6 × 5 cm tumor, which necessitated partial resection of the overlying pectoralis major muscle. Complete macroscopic tumor removal was achieved via sharp dissection around the margins, accompanied by concurrent axillary and supraclavicular lymph node dissection.

**Figure 1:**
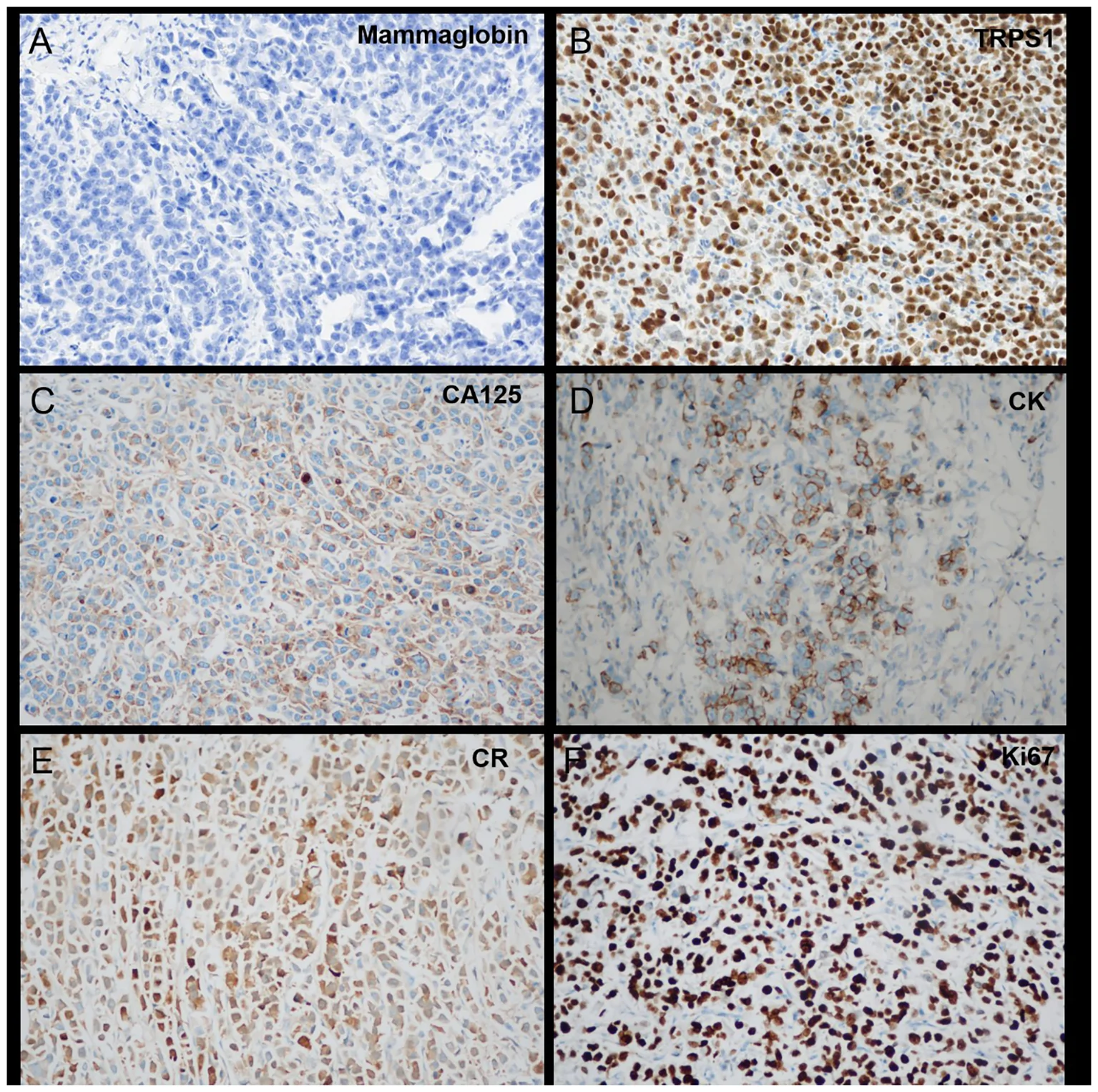
Immunohistochemical staining profiles. **(A, B)** Mammaglobin negative staining in tumor tissue and TRPS1 positive nuclear staining; **(C, D)** IHC results from outside-hospital pathology including CA125 (+), CK (partial perinuclear +), Calretinin (+),Ki67 70%. IHC scoring followed CAP-ASCO criteria. Nuclear staining was considered positive for TRPS1. Scale bars: 100 μm.

Postoperative pathological examination at that institution initially listed malignant mesothelioma as a differential diagnosis. Immunohistochemical (IHC) staining revealed the following profile: CA125 (+), CK (partial perinuclear +), S-100 (–), p53 (mutant pattern expression on IHC; sequencing proven nonsense mutation was not confirmed), GCDFP-15 (–), ER (–), PR (–), HER2 (1+), GATA-3 (–), and Myoglobin (–) (**Figure 1**). IHC interpretation followed ASCO/CAP guidelines: HER2 1+ was defined as faint incomplete membranous staining; nuclear staining was considered positive for TRPS1 and SOX10.

### 2.3 Diagnostic conundrum and pathological reassessment

Due to the ambiguous nature of the initial pathology, a consultation was sought at Qianfoshan Hospital of Shandong Province, which characterized the tumor as a high-grade epithelioid neoplasm of the chest wall with a conflicting immunoprofile: SOX10 (+), S100 (partially +), Melan-A (partially +), CK20 (partially +), BCOR (partially +), Desmin (partially +), and GFAP (–).

Upon referral to our institution, an internal pathology review confirmed a poorly-differentiated, epithelioid malignant neoplasm of uncertain lineage of uncertain lineage. Focal partial pan-cytokeratin positivity provided only suggestive but non-definitive evidence for epithelial differentiation; definitive classification as a carcinoma could not be established based on morphology and IHC alone. The differential diagnosis included carcinoma, melanoma, and sarcoma. Additional IHC for HMB45 and molecular testing for *SS18-SSX* fusion to fully rule out melanoma and synovial sarcoma could not be performed due to limited archived tumor tissue.

### 2.4 Metastatic workup and molecular profiling

**Figure 2:**
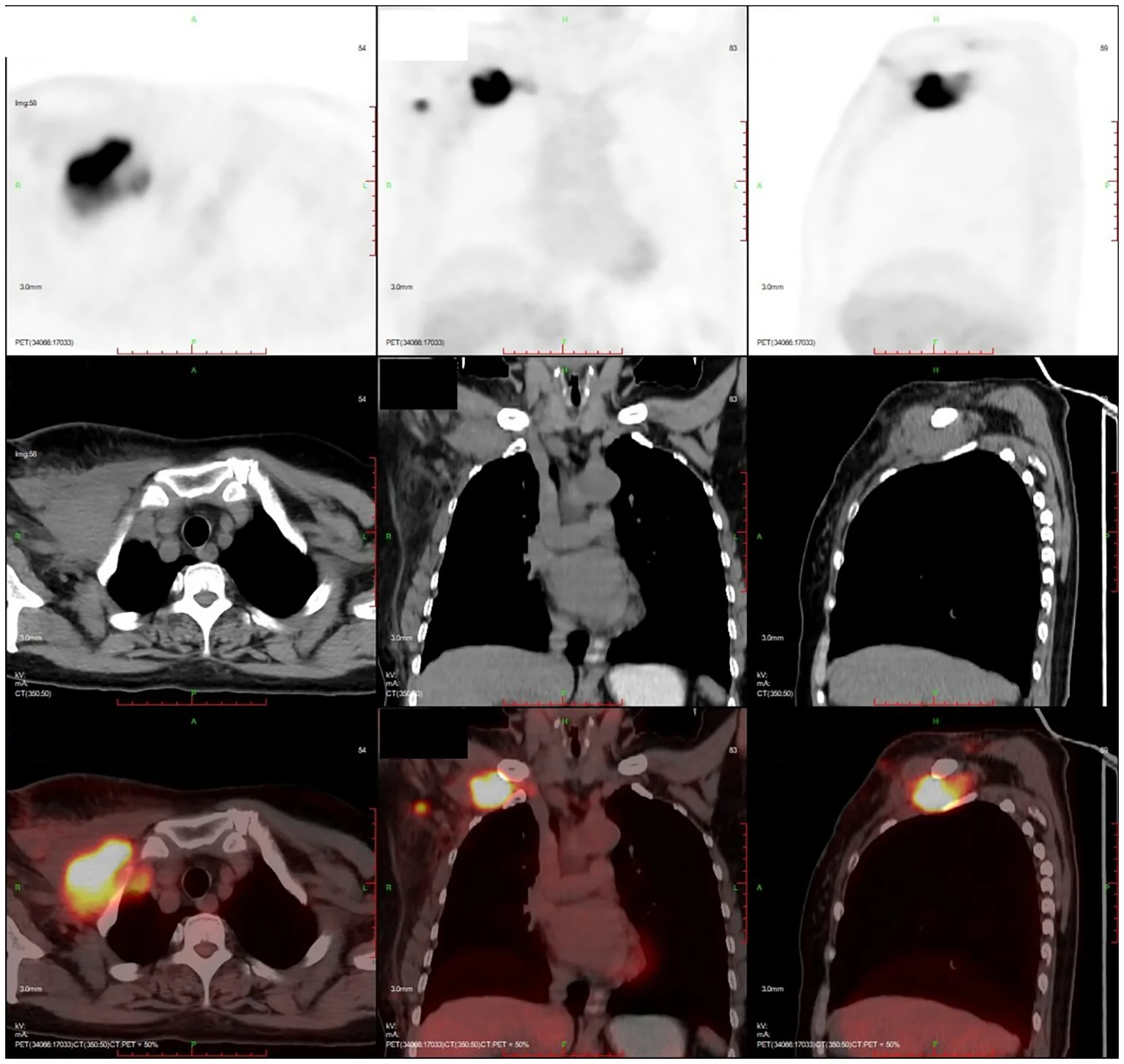
Pre-treatment PET-CT. Maximum-intensity projection and axial images demonstrating hypermetabolic lesions in right clavicular inferior soft-tissue region, right axillary, right supraclavicular and right posterior cervical lymph nodes. SUVmax values up to 21.7, consistent with metastatic disease. No definite primary tumor was identified.

Positron emission tomography-computed tomography (PET-CT) demonstrated: a hypermetabolic soft-tissue mass inferior to the right clavicle with markedly elevated activity (SUVmax ≈ 21.7), suggestive of local recurrence or residual metastasis; and enlarged hypermetabolic lymph nodes in the right axilla, right supraclavicular region, and right posterior cervical triangle (SUVmax ≈ 18.6), confirming regional lymph node involvement (**Figure 2**). Despite comprehensive imaging, no distinct primary tumor site was identified.

Of note, axillary and supraclavicular lymph-node enlargement is non-specific; it can occur in melanoma, lung cancer, lymphoma, as well as breast cancer, and nodal location alone cannot favor breast origin.

**Figure 3:**
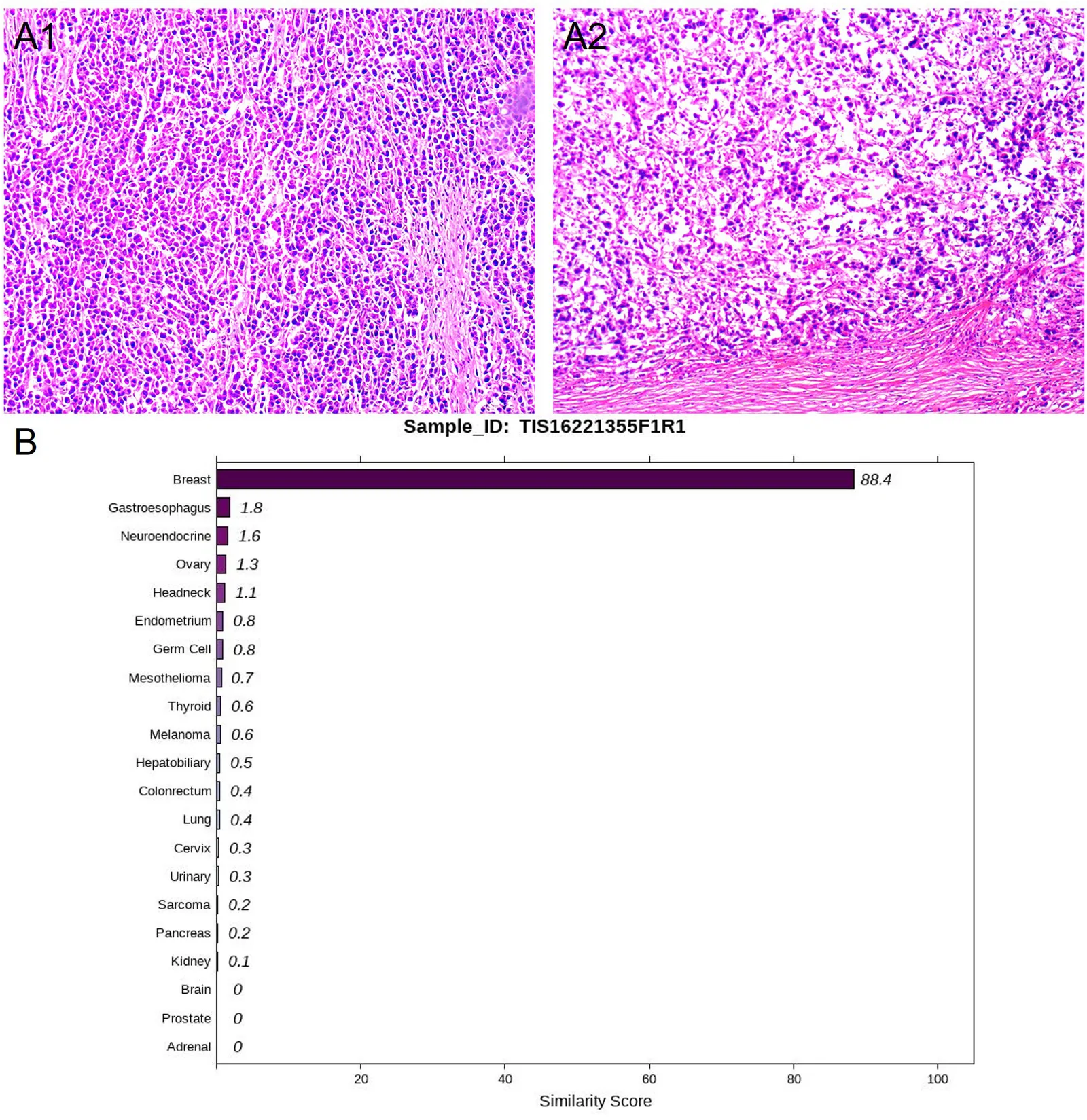
Hematoxylin and Eosin-stained pathological diagnosis and the Canhelp^®^-Origin 90-gene expression assay of resected lesions. **(A1, A2)** Hematoxylin and Eosin stained pathological diagnosis of resected lesions. **(B)** Canhelp^®^-Origin 90-gene expression assay of resected lesions. Tissue-of-origin similarity scores for 21 tumor types. The highest similarity score for breast origin was 88.4. This probabilistic molecular result, together with TRPS1-positive immunohistochemistry, supported probable metastatic triple-negative breast cancer diagnosis, acknowledging residual differential diagnostic uncertainty.

Given the lack of histopathological consensus and the occult primary, tumor origin testing via gene expression profiling, supplemented by additional IHC panels and the Canhelp^®^-Origin assay, was performed (**Figure 3**). Canhelp^®^-Origin GEP assay was performed on formalin-fixed paraffin-embedded (FFPE) surgical resection specimen of chest-wall tumor. Pre-assay quality control confirmed sufficient tumor cellularity (> 20 % tumor content), meeting manufacturer-defined sample-adequacy criteria for valid assay interpretation. The Canhelp^®^-Origin 90-gene GEP assay was performed following manufacturer-validated real-time PCR protocols, generating similarity scores across 21 tumor types; it functions as a probabilistic transcriptional classifier rather than a tool for occult lesion detection or localization. In this case, the assay output yielded a breast-origin similarity score of 88.4 (**Figure 3B**). Crucially, the integrated results demonstrated TRPS1 positivity (+) and Mammaglobin negativity (–) (**Figures 1A, B**).

The multi‑step probabilistic diagnostic reasoning was as follows, with all immunohistochemical results summarised in (**Table 1**). First, partial S100/Melan-A/SOX10 positivity raised strong differential for melanoma; partial BCOR/Desmin positivity raised synovial sarcoma differential. Nevertheless, these markers are not entirely specific, and focal partial staining pattern rather than diffuse strong staining argues against classic melanoma or synovial sarcoma. Second, TRPS1 positivity is highly sensitive for breast lesions but non-specific and cannot stand alone. Third, Canhelp^®^-Origin 90-gene GEP output gave high similarity score 88.4 for breast lineage, while scores for melanoma and sarcoma were very low (< 1.0). This molecular classifier result formed the heaviest weight of evidence favoring breast origin. Fourth, conventional breast-specific markers (GATA-3, Mammaglobin, GCDFP-15) were negative, consistent with reported TNBC cases in literature. No single marker is definitive; our conclusion remains probabilistic, not definitive.

**Table 1:** Summary of immunohistochemical markers, results and diagnostic interpretations.

Marker	Result	Diagnostic interpretation
Pan-CK	Partial perinuclear +	Focal suggestive epithelial differentiation; non-definitive for carcinoma
CA125	+	Non-specific marker
S-100	Partial +	Raises melanoma differential; partial staining not typical for classic melanoma
SOX10	+	Supports melanoma differential; also reported in subsets of TNBC
Melan-A	Partial +	Melanoma differential; partial staining atypical for melanoma
BCOR	Partial +	Synovial sarcoma differential; non-specific
Desmin	Partial +	Sarcoma/mesenchymal differential; focal staining
GFAP	−	Makes glial lineage unlikely
ER	−	Negative for hormone receptor
PR	−	Negative for hormone receptor
HER2	(1+)	Interpreted as negative per ASCO/CAP (TNBC criterion met)
GATA-3	−	Negative conventional breast marker; frequently negative in TNBC
GCDFP-15	−	Negative conventional breast marker
Mammaglobin	−	Negative conventional breast marker
TRPS1	+	High sensitivity for breast neoplasms; limited specificity, also expressed in sarcoma/melanoma subsets
Calretinin	+	Non-specific mesothelial marker
p53 IHC	Mutant-pattern expression	Abnormal tumor suppressor expression; cannot confirm sequencing-proven nonsense mutation

No single marker yields definitive diagnosis. Partial/focal staining patterns reduce confidence in melanoma/sarcoma, but these diagnoses cannot be fully excluded given absence of HMB45 and SS18-SSX fusion testing. Probabilistic favoring of breast origin relies primarily on Canhelp^®^-Origin GEP similarity score of 88.4.

PD L1, TMB, MSI status and comprehensive genomic profiling (NGS CGP) were not performed. Immune checkpoint inhibitor therapy was administered empirically.

Taking together the 88.4 similarity score favoring breast origin, TRPS1 positivity, negative conventional breast associated markers and clinical imaging features, we reached a probabilistic, integrated diagnosis of probable metastatic triple negative breast cancer (ER (–), PR (–), HER2 (–); HER2 (1+) interpreted as negative per ASCO/CAP criteria). Melanoma and synovial sarcoma remained important differential diagnoses that could not be definitively excluded.

### 2.5 Treatment regimen and early response

The patient was initiated on combination chemotherapy with the AC regimen (Adriamycin and Cyclophosphamide) plus an anti PD 1 immune checkpoint inhibitor (tislelizumab). Dosing schedule: Adriamycin 60 mg/m² intravenously day 1, Cyclophosphamide 600 mg/m² intravenously day 1, serplulimab 300 mg intravenously day 1, repeated every 21 days for a planned total of six cycles. This systemic regimen was selected via multidisciplinary tumor-board discussion, guided by the probabilistic diagnosis of metastatic TNBC and patient performance status. After two cycles, restaging CT revealed a marked reduction in the size of the axillary and cervical lymph nodes compared to baseline. Per RECIST 1.1 criteria, the response was assessed as a partial response (PR).

### 2.6 Locoregional radiotherapy and maintenance therapy

Following the fourth cycle of systemic therapy, consolidative radiotherapy was initiated on May 7, 2025. A total dose of 60 Gy was delivered to the gross tumor volume (GTV), and 50 Gy to the clinical target volume (CTV)—which encompassed the right axilla, right neck, and right breast region—administered in 28 fractions, with completion on June 11, 2025. Concurrent chemo-immunotherapy was continued. After completing six cycles of the combination regimen, due to limited patient financial resources, the anti-PD-1 agent was switched to sintilimab 200 mg intravenously every 21 days. Maintenance therapy consisting of oral capecitabine 1500 mg/m² twice daily days 1–14 every 21 day cycle combined with sintilimab 200 mg intravenously every 21 days. Maintenance capecitabine plus immune checkpoint inhibitor was selected referencing real world clinical evidence for advanced stage triple negative breast cancer after response to first line chemo immunotherapy, to sustain disease control in this high risk metastatic setting.

### 2.7 Final outcome and complete remission

**Figure 4:**
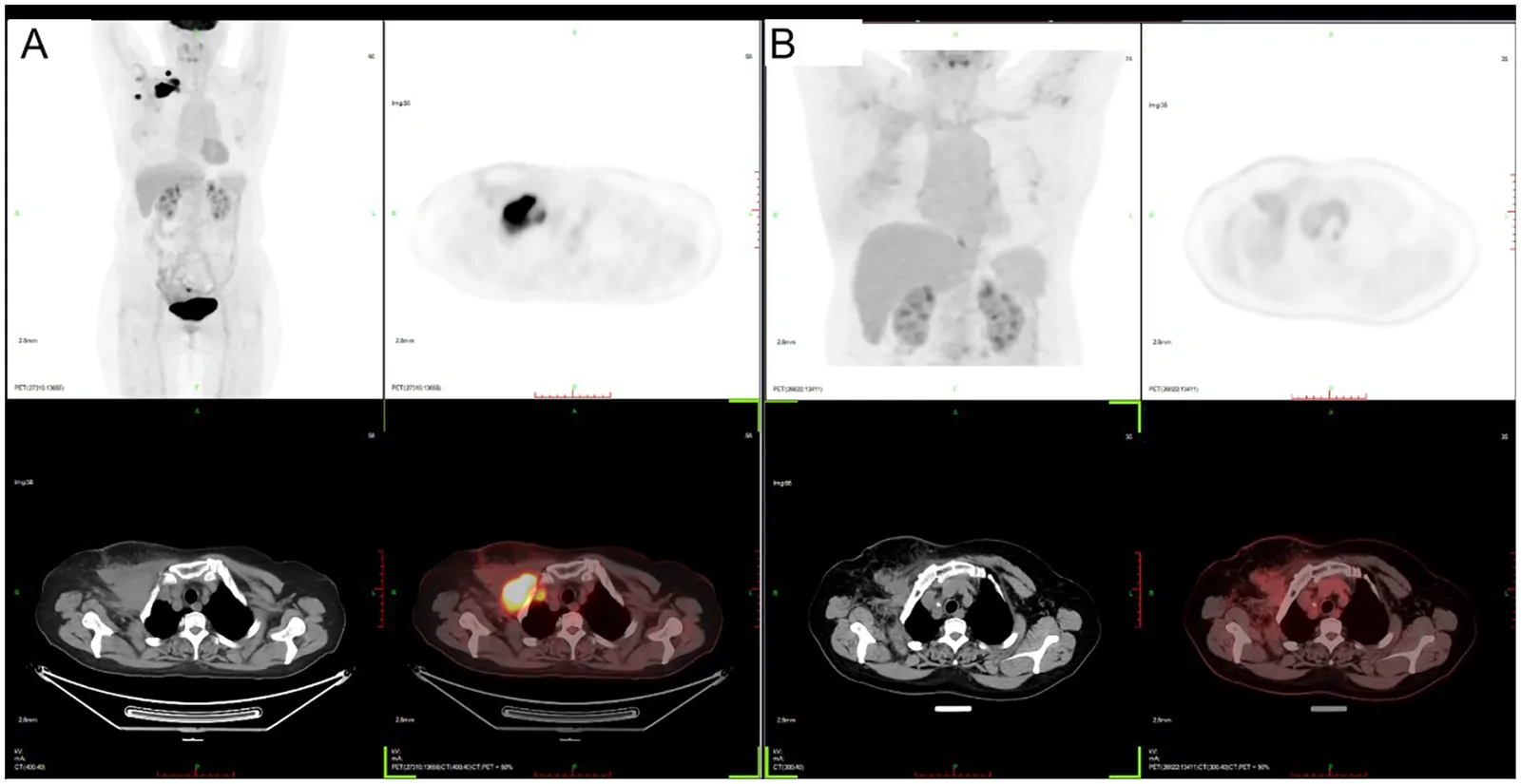
Post-treatment follow-up PET-CT (January 15, 2026): **(A)** were before treatment, **(B)** were PET/CT scan performed on January 15, 2026 (the last follow-up images). Complete metabolic resolution of previous enlarged lymph nodes in right axillary and cervical regions. No residual hypermetabolic tumor lesions, meeting RECIST 1.1 criteria for complete response (CR).

On January 15, 2026, a response-assessment PET-CT revealed complete resolution of the previously enlarged lymph node located posterior to the pectoralis major muscle in the right axillary region, with no detectable metabolic activity in the residual soft tissue, indicating effective tumor control and absence of residual disease. According to RECIST 1.1 criteria, the patient was classified as having achieved a complete response (CR) (**Figure 4**). Nevertheless, this complete radiological response cannot serve as retrospective confirmation of tumor tissue of origin; anthracycline based chemotherapy and immunotherapy can induce responses in multiple chemosensitive malignancies including synovial sarcoma and melanoma. The maintenance therapy with capecitabine and immunotherapy is currently ongoing, with good tolerance.

### 2.8 Ethics and patient consent

This single patient case report was performed in accordance with the Declaration of Helsinki. Written informed consent was obtained from the patient for publication of de identified clinical data, pathological slides and imaging examinations. No identifiable personal information has been presented within this manuscript.

## 3 Discussion

We present a case illustrating the diagnostic and therapeutic approach to cancer of unknown primary (CUP). CUP represents a heterogeneous group of malignant neoplasms characterized by the presence of metastatic disease in the absence of an identifiable primary tumor site, despite thorough clinical, pathological, and radiological evaluation (4). CUP is frequently associated with a minute primary tumor or spontaneous regression, which obscures the histogenetic origin of the malignancy. As a result, clinical management is highly complex. Although ESMO, NCCN and Chinese domestic guidelines exist for CUP management, high-level evidence for unfavorable-risk CUP subgroups remains limited.

To address this clinical challenge, a standardized diagnostic algorithm has been widely adopted in routine practice. It encompasses a comprehensive evaluation comprising detailed history taking, systematic physical examination, targeted laboratory investigations, and multimodality imaging—including contrast-enhanced CT, MRI, and ^18^F-FDG PET/CT. At the histopathological level, immunohistochemistry (IHC) serves as a cornerstone tool for tumor origin attribution, enabling inference of likely tissue lineage through the detection of lineage-specific protein markers. However, in many poorly differentiated or undifferentiated tumors, the staining patterns of IHC are often atypical or contradictory, and can only provide clear clues for approximately 35% to 55% of cases (5). Metastatic cancer cells largely retain the gene expression profiles of their tissue of origin, enabling the inference of primary site through analysis of specific gene expression patterns in tumor tissue. Consequently, gene expression profiling (Canhelp^®^-Origin) has emerged as a valuable diagnostic tool for identifying the likely origin in CUP.

Multiple tissue-of-origin classifier platforms are currently available in clinical practice, including CancerTYPE ID 92-gene assay, DNA-methylation-based classifiers, whole-transcriptome RNA-sequencing classifiers and artificial-intelligence-assisted pathological diagnostic tools (6, 7). Canhelp^®^-Origin represents one validated assay among these diagnostic modalities. It should be noted that assay interpretability rate reflects the proportion of samples yielding valid output, and is not equivalent to diagnostic accuracy verified against gold-standard reference diagnosis. Canhelp^®^-Origin is a 90-gene expression profiling assay specifically developed for the differential diagnosis of 21 common malignant neoplasms. The panel includes carcinomas of the breast, lung, colorectum, prostate, ovary, and pancreas, as well as adrenal, renal, thyroid, hepatic, endometrial, cervical, and gastroesophageal cancers, along with neuroendocrine tumors. This assay is a real-time PCR–based method that utilizes differential gene expression profiles to classify tumors into one of 21 lineage-specific tumor types, achieving an accuracy of 94.4% and a specificity greater than 99% (8, 9). These performance metrics originate from validation cohorts with known primary tumors and may not directly translate to real world CUP patients where true primary sites cannot be confirmed. This capability to identify the tissue of origin of tumors has received regulatory authorization through the European Conformity (CE) marking and approval by the National Medical Products Administration (NMPA) of China.

Furthermore, multiple studies have demonstrated that the 90-gene expression profile exhibits high diagnostic performance in challenging tumor cases, including triple-negative breast cancer, brain metastases, liver metastases, and multiple primary malignancies, with reported accuracy ranging from 92.0% to 97.4% (10). Especially in triple-negative breast cancer (TNBC), the analysis results of 90 gene expression features showed an overall consistency of 97.4% with the reference diagnosis. Genes such as *AZGP1*, *KRT19*, and *PIGR* were identified as having differential expression between TNBC and other tumor types, suggesting their potential as distinguishing markers (11). Thus, the 90-gene expression test is helpful in detecting occult lesions and guiding subsequent treatment. The prognosis of CUP patients largely depends on the understanding of the biological characteristics of the primary tumor. Studies have shown that CUP patients who receive tumor type-specific treatment have a better median survival period (12.5 months vs. 9.1 months) than those who receive empirical treatment (12). Therefore, clarifying the tissue origin of the tumor and adopting targeted treatment is of great significance for improving the prognosis of patients. And Canhelp^®^-Origin helps to clarify the source of the primary tumor, providing significant assistance in the diagnosis and treatment of CUP.

In the clinical pathological diagnosis of breast cancer, especially when dealing with metastatic lesions or special subtypes of breast cancer, the core challenge lies in finding immunohistochemical (IHC) markers with high sensitivity and specificity. Currently, traditional markers such as estrogen receptor (ER), GATA3, GCDFP-15 and mammaglobin, although widely used, may have many limitations due to their limited specificity. TRPS1 (Tricho-rhino-phalangeal syndrome type 1) has emerged as a novel biomarker for breast cancer. It exhibits an extremely high expression rate in breast cancer tissues, with both its mRNA and protein levels significantly higher in normal and tumor breast tissues compared to other human tissues. The high sensitivity of TRPS1 in breast cancer has been confirmed by multiple large-scale studies. A tissue microarray study involving 19,201 tumor samples revealed that the positive rate of TRPS1 in various types of breast cancer ranged from 51% to 100%, significantly higher than that of the traditional marker GATA3. Recent studies have consistently shown that TRPS1 is highly expressed in breast cancer tissues, particularly demonstrating outstanding diagnostic sensitivity in the most challenging-to-differentiate triple-negative breast cancer (TNBC) (13, 14). Studies have demonstrated that TRPS1 is positively expressed in over 90% of TNBC cases (13). Furthermore, its positive expression rate exceeds 83% across various histological subtypes, including invasive carcinoma of no special type (NST), metaplastic carcinoma, and medullary carcinoma (15). The positive rate of TRPS1 in metastatic breast cancer (99.4%) was also higher than that of GATA3 (90.9%, p < 0.001) (16). Moreover, TRPS1 is stably expressed in various types of specimens, greatly expanding its clinical application scenarios. Although TRPS1 has outstanding sensitivity, its specificity is limited. Studies have shown that TRPS1 is expressed in 86 types of non-breast tumors, including soft tissue tumors (with a positive rate as high as 100%), salivary gland tumors (46%), squamous cell carcinomas (35%), and gynecological tumors (40%) (17). It is worth noting that TRPS1 shows nuclear positive expression in sweat gland papilloma, which is consistent with the theory of its origin from breast-like glands, but also suggests its potential cross-reaction in non-breast cancers (18). Therefore, the sole use of TRPS1 may lead to misdiagnosis and it is necessary to conduct a comprehensive assessment in combination with other markers (such as GATA3 and SOX10).

SOX10 (SRY-box containing gene 10), as a member of the SOX family, is another important biomarker that has shown significant value in breast cancer, especially in the TNBC subtype. Given the distinct sensitivity and specificity profiles of TRPS1 and SOX10, these two markers exhibit complementary diagnostic roles. In a study involving 84 cases of triple-negative breast cancer (TNBC), combined evaluation of their expression revealed that 52.4% of cases were positive for both markers [SOX10(+)/TRPS(1+)], 34.5% were TRPS1-positive but SOX10-negative [SOX10 (–)/TRPS(1+)], 9.5% were SOX10-positive but TRPS1-negative [SOX10(+)/TRPS1 (–)], and only 3.6% were negative for both [SOX10 (–)/TRPS1 (–)] (15). In our case, the immunohistochemistry of the patient indicated SOX-10 (+), TRPS1 (+), and HER2 (1+)., interpreted as negative according to ASCO/CAP criteria. Notably, concurrent SOX10 positivity raised important differential diagnostic possibilities including melanoma and synovial sarcoma. The final probabilistic diagnosis was not built on nodal metastatic site, but synthesized from the (Canhelp^®^-Origin) similarity score of 88.4 favoring breast origin and supportive TRPS1 staining, while acknowledging residual diagnostic uncertainty.

To the best of our knowledge, this is one of the first reports to detail the successful diagnostic workup of a CUP patient presenting with predominant axillary-regional lymph-node involvement alongside a primary-site chest-wall mass, wherein the integration of a 90-gene GEP assay (Canhelp^®^-Origin) with the novel IHC markers TRPS1 and SOX10 was pivotal in establishing a diagnosis of metastatic TNBC and guiding effective targeted therapy (15). This case underscores the practical utility of this combined molecular-pathological approach in resolving diagnostically challenging CUP cases, while highlighting inherent diagnostic limitations when facing conflicting immunophenotypes (19).

In conclusion, this case successfully provided a probable diagnosis for a case of axillary metastatic cancer of unknown primary origin by integrating the detection of tissue-origin genes with specific immunohistochemical markers, and based on this, a targeted treatment plan was selected, achieving a good clinical outcome. This once again highlights the significant value of TRPS1 as a biomarker for the differential diagnosis of breast cancer, especially triple-negative breast cancer. Meanwhile, this case demonstrates that the tissue-origin gene test (Canhelp^®^-Origin) can effectively assist clinical decision-making, particularly providing a new diagnostic approach for CUP, with full awareness of its probabilistic nature and diagnostic limitations, thereby improving treatment options and prognosis for such patients.

## Data Availability

The original contributions presented in the study are included in the article/supplementary material. Further inquiries can be directed to the corresponding author.

## References

[1] Qaseem A Usman N Jayaraj JS Janapala RN Kashif T . Cancer of unknown primary: a review on clinical guidelines in the development and targeted management of patients with the unknown primary site. Cureus. (2019) 11:e5552. doi: 10.7759/cureus.5552 31695975 PMC6820325

[2] Ren M Cai X Jia L Bai Q Zhu X Hu X . Comprehensive analysis of cancer of unknown primary and recommendation of a histological and immunohistochemical diagnostic strategy from China. BMC Cancer. (2023) 23:1175. doi: 10.1186/s12885-023-11563-1 38041048 PMC10691136

[3] Georgescu AC Georgescu TA Duca-Barbu SA Pop LG Toader DO Suciu N . A comprehensive review of Trps1 as a diagnostic immunohistochemical marker for primary breast carcinoma: latest insights and diagnostic pitfalls. Cancers. (2024) 16:3568 doi: 10.3390/cancers16213568 39518009 PMC11545765

[4] Dermawan JK Rubin BP . The role of molecular profiling in the diagnosis and management of metastatic undifferentiated cancer of unknown primary(☆): molecular profiling of metastatic cancer of unknown primary. Semin Diagn Pathol. (2021) 38:193–8. doi: 10.1053/j.semdp.2020.12.001 33309276

[5] Hainsworth JD Greco FA . Gene expression profiling in patients with carcinoma of unknown primary site: from translational research to standard of care. Virchows Archiv Int J Pathol. (2014) 464:393–402. doi: 10.1007/s00428-014-1545-2 24487792

[6] Raghav K Overman M Poage GM Soifer HS Schnabel CA Varadhachary GR . Defining a distinct immunotherapy eligible subset of patients with cancer of unknown primary using gene expression profiling with the 92-gene assay. Oncologist. (2020) 25:e1807–11. doi: 10.1634/theoncologist.2020-0234 32893931 PMC7648339

[7] Ghani H Helmstetter A Ribeiro JR Maney T Rock S Feldman RA . Gpsai: a clinically validated AI tool for tissue of origin prediction during routine tumor profiling. Cancer Res Commun. (2025) 5:1477–89. doi: 10.1158/2767-9764.Crc-25-0171 40741943 PMC12399951

[8] Sun W Wu W Wang Q Yao Q Feng Q Wang Y . Clinical validation of a 90-gene expression test for tumor tissue of origin diagnosis: a large-scale multicenter study of 1417 patients. J Transl Med. (2022) 20:114. doi: 10.1186/s12967-022-03318-6 35255924 PMC8900384

[9] Ye Q Wang Q Qi P Chen J Sun Y Jin S . Development and clinical validation of a 90-gene expression assay for identifying tumor tissue origin. J Mol Diagnostics JMD. (2020) 22:1139–50. doi: 10.1016/j.jmoldx.2020.06.005 32610162

[10] Zheng Y Ding Y Wang Q Sun Y Teng X Gao Q . 90-gene signature assay for tissue origin diagnosis of brain metastases. J Transl Med. (2019) 17:331. doi: 10.1186/s12967-019-2082-1 31570099 PMC6771090

[11] Wang Q Xu M Sun Y Chen J Chen C Qian C . Gene expression profiling for diagnosis of triple-negative breast cancer: a multicenter, retrospective cohort study. Front Oncol. (2019) 9:354. doi: 10.3389/fonc.2019.00354 31134153 PMC6513966

[12] Hainsworth JD Rubin MS Spigel DR Boccia RV Raby S Quinn R . Molecular gene expression profiling to predict the tissue of origin and direct site-specific therapy in patients with carcinoma of unknown primary site: a prospective trial of the Sarah Cannon Research Institute. J Clin Oncol Off J Am Soc Clin Oncol. (2013) 31:217–23. doi: 10.1200/jco.2012.43.3755 23032625

[13] McIntire PJ Duckworth LA Van Arnam J Abdelwahab H Shin SJ . Trps1, a new promising marker for assessment of distant metastatic breast cancer. Adv Anatomic Pathol. (2023) 30:380–7. doi: 10.1097/pap.0000000000000409 37593968

[14] Hashmi AA Brogi E Wen HY . Trichorhinophalangeal syndrome type 1 (Trps1) in breast pathology: diagnostic utility and pitfalls. Diagn Pathol. (2025) 20:26. doi: 10.1186/s13000-025-01623-4 40025593 PMC11872298

[15] Elgohary SA Elmaraghy MN Nada OH Farid RM Elshaarawi S Ashoush BM . The diagnostic significance of immunohistochemical expression of Sox10 and Trps1 in triple negative breast cancer. Ann Diagn Pathol. (2025) 78:152480. doi: 10.1016/j.anndiagpath.2025.152480 40245691

[16] Wu Y Chen F Pan L Chao X Li M Luo R . Diagnostic utility and sensitivities of matrix Gla protein (Mgp), Trps1 and Gata3 in breast cancer: focusing on metastatic breast cancer, invasive breast carcinoma with special features, and salivary gland-type tumours. Pathology. (2024) 56:516–27. doi: 10.1016/j.pathol.2024.01.003 38570266

[17] Lennartz M Löhr N Höflmayer D Dwertmann Rico S von Bargen C Kind S . Trps1 is a highly sensitive marker for breast cancer: a tissue microarray study evaluating more than 19,000 tumors from 152 different tumor entities. Am J Surg Pathol. (2024) 48:637–51. doi: 10.1097/pas.0000000000002213 38647255 PMC11093513

[18] Velthof L Van Dorpe J Tummers P Creytens D Van de Vijver K . Trps1 is consistently expressed in hidradenoma papilliferum. Int J Gynecological Pathol Off J Int Soc Gynecological Pathologists. (2025) 44:99–103. doi: 10.1097/pgp.0000000000001042 38959400

[19] Qi P Sun Y Pang Y Liu J Cai X Huang S . Diagnostic utility of a 90-gene expression assay (Canhelp-Origin) for patients with metastatic cancer with an unclear or unknown diagnosis. Mol Diagnosis Ther. (2025) 29:81–9. doi: 10.1007/s40291-024-00746-6 39333459

